# Flight Morphology, Compound Eye Structure and Dispersal in the Bog and the Cranberry Fritillary Butterflies: An Inter- and Intraspecific Comparison

**DOI:** 10.1371/journal.pone.0158073

**Published:** 2016-06-23

**Authors:** Camille Turlure, Nicolas Schtickzelle, Hans Van Dyck, Brett Seymoure, Ronald Rutowski

**Affiliations:** 1 Université catholique de Louvain, Biodiversity Research Centre, Earth and Life Institute, Louvain-la-Neuve, Belgium; 2 School of Life Sciences, Arizona State University, Tempe, Arizona, United States of America; Oxford Brookes University, UNITED KINGDOM

## Abstract

Understanding dispersal is of prime importance in conservation and population biology. Individual traits related to motion and navigation during dispersal may differ: (1) among species differing in habitat distribution, which in turn, may lead to interspecific differences in the potential for and costs of dispersal, (2) among populations of a species that experiences different levels of habitat fragmentation; (3) among individuals differing in their dispersal strategy and (4) between the sexes due to sexual differences in behaviour and dispersal tendencies. In butterflies, the visual system plays a central role in dispersal, but exactly how the visual system is related to dispersal has received far less attention than flight morphology. We studied two butterfly species to explore the relationships between flight and eye morphology, and dispersal. We predicted interspecific, intraspecific and intersexual differences for both flight and eye morphology relative to i) species-specific habitat distribution, ii) variation in dispersal strategy within each species and iii) behavioural differences between sexes. However, we did not investigate for potential population differences. We found: (1) sexual differences that presumably reflect different demands on both male and female visual and flight systems, (2) a higher wing loading (i.e. a proxy for flight performance), larger eyes and larger facet sizes in the frontal and lateral region of the eye (i.e. better navigation capacities) in the species inhabiting naturally fragmented habitat compared to the species inhabiting rather continuous habitat, and (3) larger facets in the frontal region in dispersers compared to residents within a species. Hence, dispersers may have similar locomotory capacity but potentially better navigation capacity. Dispersal ecology and evolution have attracted much attention, but there are still significant gaps in our understanding of the mechanisms of dispersal. Unfortunately, for many species we lack detailed information on the role of behavioural, morphological and physiological traits for dispersal. Our novel study supports the existence of inter- and intra-specific evolutionary responses in both motion and navigation capacities (i.e. flight and eye morphology) linked to dispersal.

## Introduction

Dispersal is defined as the movement of individuals or propagules that can sustain gene flow among local populations [1]. Understanding the dispersal process is of prime importance in conservation biology, especially to guide the creation and management of nature reserves and threatened species in fragmented landscapes [2–4], and of conceptual significance, in population biology and evolutionary biology [5,6]. Following the view of Nathan *et al*. [7], dispersal (and actually any type of movement, including migration and foraging) can be studied within a single, new conceptual framework: the movement ecology paradigm [8]. This conceptual framework focuses on four main questions: i) *why* disperse, ii) *how* to disperse, iii) *when* and *where* to disperse and iv) *what* are the *consequences* of dispersal at both ecological and evolutionary levels?

The decision to disperse from a (natal) habitat patch (i.e. *why* disperse?) may be context- and condition-dependent. For example, the density of conspecifics and/or kin in a habitat patch may stimulate emigration to limit competition [9,10]. In actively dispersing organisms, successfully travelling across the landscape (i.e. *how*, *when* and *where* to disperse?) requires time and energy, but also efficient motion and effective navigation capacities [11]. Motion capacities refer to the “ability to move in various ways or modes” [7]. These capacities vary with morphology (e.g. dispersal dimorphism) and physiology (e.g. energy metabolism) both within and between species. Navigation capacities refer to the “ability to orient in space and/or time, selecting where […] and/or when […] to move” [7]. They are linked to sensory abilities and spatial cognitive abilities (through organs, such as eyes for vision, nose and antennae for olfaction, and the brain for celestial navigation and cognitive maps)(e.g. [12]). Both motion and navigation capacities may affect and be affected by dispersal. In flying organisms, wing size and wing loading (i.e. ratio of body mass to wing area) are associated with long-distance movements [13,14]. High wing loading is associated with fast flight [15], but it may also relate to other flight performance measures like manoeuvrability, which may be more important in the context of mate-locating behaviour than dispersal per se [16]. In birds, individuals of the migrant phenotypes have been identified as that with a more efficient metabolism that decreases the physiological cost of movement [17]. In migratory Monarch butterflies, both the cells in the central brain and the antennae are used to orient flight during migration (e.g. using sun or polarization compass orientation; [18]).

Traits that relate to motion and navigation are expected to evolve relative to the dispersal propensity of a species (or population), and thus, to be influenced by habitat and landscape features. Dispersal-related traits may differ at the intra- and interspecific level. (1) Individuals from different species may have to deal with spatially different habitats or resource distributions, and less energy as well as lower navigation capacities are arguably needed for effective movement through fine-grained, continuous habitat than between coarse-grained, separated habitat patches. (2) Also, individuals from populations that experience higher levels of habitat fragmentation will have to travel greater distances between habitat patches. (3) Furthermore, dispersal-related traits may differ among conspecific individuals that exhibit different dispersal strategies (e.g. disperser vs. resident; [19,20]). (4) Lastly, dispersal differences, specifically in flight and sensory morphology, between males and females may arise due to differences in sex-specific activities. It relates to frequently observed sexual dimorphism in flight morphology (to maintain flight performance with egg load in females on the one hand and mate location behaviour in males on the other; [16]) and eye morphology such as larger facets in the frontal region of the eyes of males to track females vs. larger facets in the ventral region of the eyes of females to spot suitable egg-laying sites.

Insects, and butterflies in particular, respond quickly to environmental change and have been used for decades to study the effects of environmental change [21] on animal behaviour including dispersal. Some studies focus on the impact of environmental change on butterfly morphology and physiology, as key indicators of motion capacities. Evolutionary changes in flight morphology and physiology have, for example, been related to range expansions, colonization success, habitat fragmentation and migration [22–25]. In the study presented here, we address how the butterfly visual system varies with habitat distribution and individual dispersal strategy. In butterflies, the visual system is a key contributor to their navigation capacities. Spatial resolution and visual sensitivity of their compound eyes vary as inferred from studies of variation in eye size, facet size and interommatidial angle in different regions of the eye [26–28]. Variation in eye structure is expected to relate to broad scale movement patterns because of the critical role eye structure has in extracting information from the environment, but its relation with dispersal ability is largely unknown.

We studied two butterfly species, *Boloria eunomia* and *B*. *aquilonaris*, and explored potential links between dispersal and both motion and navigation capacities by focusing on adult flight morphology and compound eye structure. These species were chosen due to significant changes and differences in the historical and current distributions of their respective habitats [29], which in turn, might lead to different selection regimes on dispersal-related traits. In Belgium, the habitat patches of *B*. *aquilonaris* and therefore their populations have been fragmented for decades, whereas *B*. *eunomia* habitat patches and their populations are more continuously distributed along rivers. Accordingly, the two species differ in dispersal propensity. The analyses of genetic structure within the same landscape using microsatellite markers revealed that local populations were connected by consistent gene flow at each generation (unpublished results). Therefore, dispersal between habitat patches occurs in both species, even between the most separated local populations of *B*. *aquilonaris*. Moreover, intensive Mark-Release-Recapture studies performed on both species at a regional scale in southern Belgium revealed that the maximum distance recorded between successive captures was 6–7 km for *B*. *eunomia* ([30] and unpublished data) and up to 37 km for *B*. *aquilonaris* ([31] and unpublished data).

Therefore, our specific hypotheses and predictions concerning flight morphology and eye structure are as follows. First, if selection acts differently on the behaviour of males and females in both species, we expect a similar sexual dimorphism in flight and eye morphology in both species associated with the contrasting lifestyles of males and females. Males of both species adopt a patrolling strategy to locate females (i.e., males spend most of their active time flying in search of and chasing females. In contrast females allocate their time to feeding, resting and searching for egg-laying sites. Accordingly, compared to females, males are predicted to have bigger eyes with larger facets for female detection, a key determinant of mating success. We also predict that males will have higher wing loading to facilitate more rapid manoeuvrable flight [16]. Second, focusing on the effect of habitat distribution, we expect interspecific differences in flight and eye morphology. To cover longer dispersal distances, *B*. *aquilonaris* individuals are predicted to have larger relative thorax size (i.e. more flight muscles) and/or higher wing loading [32] and to have visual system features (i.e. higher eye sensitivity and/or acuity) enabling them to orient more easily in a more complex, spatially distributed habitat patch system compared to *B*. *eunomia*(cf. [28]). Note that the relationship between wing loading and dispersal is unclear in the literature [33]. Wing loading is a metric that captures the lift associated with wing size and the burden associated with body mass to infer an organism’s flying ability. Low values would indicate less energy to flight (e.g. [34,35]), but if flight speed is important to cover inter-patch distances then there can be a positive relationship between wing loading and dispersal (i.e. through fast flight; [15]). Turlure et al. [32] reported a positive correlation between dispersal movements and wing loading for the focal species in this study. Third, populations of the two species are not homogeneously distributed in the landscape resulting in populations with different levels of connectivity. However, the current experiment was not designed to investigate specifically population differences. Finally, our study did test similar predictions at the intraspecific level by comparing flight and eye morphologies between dispersers (i.e. individuals that moved out of their natal habitat patch) and residents (i.e. individuals that stayed in their natal patch) within *B*. *eunomia*. In the same vein, we predict dispersers to have larger thoraxes, higher wing loading, more (and hence) smaller facets in general but actually larger facets in the frontal region of the eye. To the best of our knowledge, these relationships have not yet been studied in flying insects.

## Materials and Methods

### Study species and morphological data collection

The bog fritillary, *Boloria eunomia*, and the cranberry fritillary, *B*. *aquilonaris*, are habitat specialists, with populations occurring in spatially discrete habitat patches in the same landscape [36]. *B*. *eunomia* inhabits bogs and wet meadows along rivers where its only host plant (*Persicaria bistorta*, the bistort) grows. These bogs and meadows are more or less continuously distributed in open areas, along rivers, which represents a case of fine-grained habitat distribution. *B*. *aquilonaris* inhabits bogs where its only host plant (*Vaccinium oxycoccos*, the cranberry) grows; those bogs have been fragmented in Belgium for decades and are usually embedded in a forest matrix, creating a coarser-grained habitat distribution. For example, the distance to the nearest neighbour habitat patch in our study landscape (Plateau des Tailles, Belgian Ardenne; N 50°24—E 5°77) is on average 1260 m (min = 443 m, max = 4525 m) for *B*. *eunomia* and 3375 m (min = 940 m, max = 6969 m) for *B*. *aquilonaris*.

**Fig 1:**
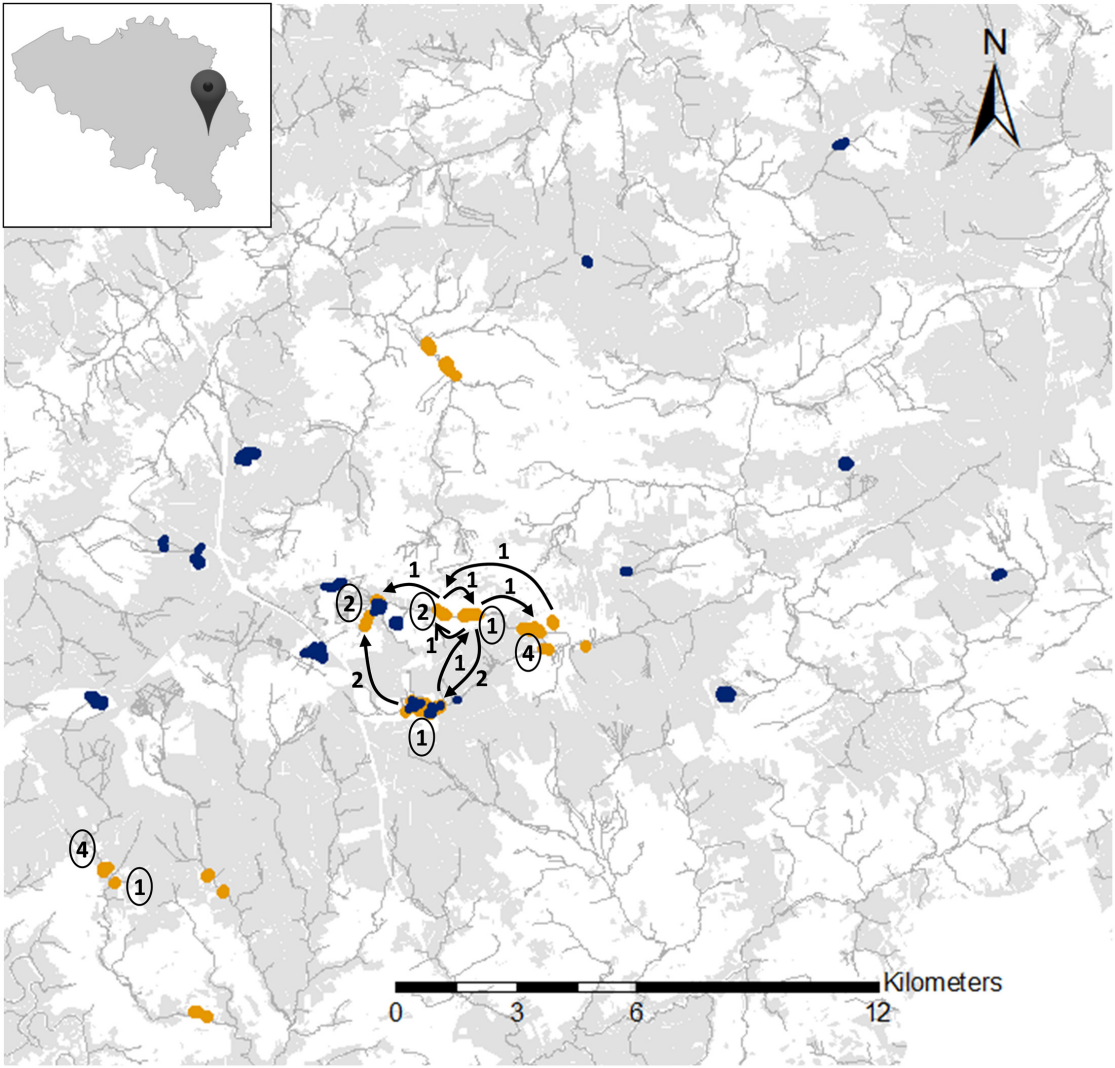
Map of the studied populations of both *Boloria* butterfly species in south-eastern Belgium (insert). Dark blue areas = *B*. *aquilonaris* populations. Light orange areas = *B*. *eunomia* populations. Black arrows and corresponding numbers = direction and number of movements of the *B*. *eunomia* dispersers. Circled numbers = Location and number of *B*. *eunomia* residents.

In the summer of 2011, we monitored 14 populations of *B*. *eunomia* and 14 populations of *B*. *aquilonaris* in the Plateau des Tailles area (Fig 1) using Mark-Release-Recapture (MRR) sampling. During each species’ flight period, all populations were visited every other day (weather permitting) and individuals were caught and marked on the underside of the left hindwing with a permanent blue pen (Stabilo OHPen universal S) using a unique alphanumeric code (a letter coding for the site and a number coding for the individual). For each (re)capture, we recorded the individual’s code and sex, and the date and population location. Using the MRR data, we assigned each individual to one of three categories: disperser (*i*.*e*. individual recaptured in a population different from the population of initial capture) or resident (*i*.*e*. individual recaptured regularly in its population of initial capture for > 12 days) or unclassified (*i*.*e*. individual that did not fit the previous categories). Dispersers and residents, and a sample of unclassified individuals, were collected to analyse flight and eye morphology. Wings were preserved in paper envelopes and heads in microtubes filled with ethanol. We refer to this set of individuals as Group1.

At the beginning of the flight period, 10 newly emerged individuals per species, sex and population were captured. Size measures were taken directly in the field with callipers. We refer to the individuals of this sample as Group2.

Site access and a permission to study the species in the field were granted and all research presented here was conducted in accordance with the laws and rules set forth by the Service Public de Wallonie.

### Morphological measurements

**Fig 2:**
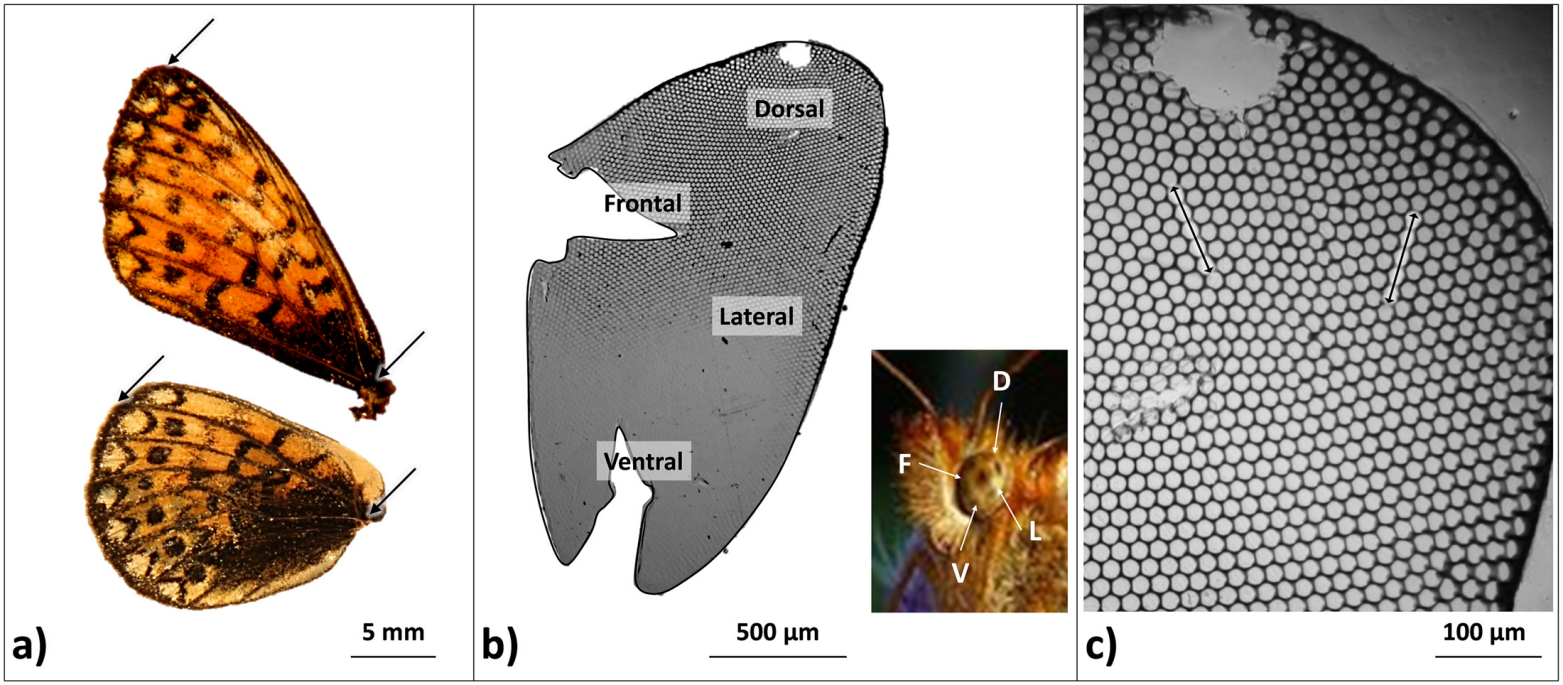
Pictures of left wings (a), left eye cornea (b) and dorsal area facets (c) from a *B*. *eunomia* female. Arrows in panel (a) indicate points between which wing length was measured: base and end of the R4 and M1 veins. In panel (b) we indicated the four different regions of the eye on the butterfly head (right picture) and on the cornea (i.e. D: dorsal, F: frontal, L: lateral and V: ventral regions) and an illustration of the way the eye area was measured (black line surrounding the four regions of the eye). In panel (c), we indicated two examples of measures of facet size, quantified on rows of five facets.

For each individual of Group1, we first measured wing length (from the base to the end of the R4 and M1 veins of the forewing and the hindwing, respectively; Fig 2a) and the area of the left forewing and the left hindwing from digital images (using a Canon EOS 5D Mark II camera). These four measures were highly correlated (Pearson correlation tests; r = 0.91–0.96, all *P* < 0.0001). Because only forewing length could be measured for all specimens, this variable was the one used in further analysis. Second, the head of each specimen that was initially preserved in ethanol was soaked in a 20% aqueous NaOH solution for 15 h to soften the tissues of the eye. The cornea was then removed and mounted in glycerol between a glass slide and coverslip following techniques used by Ziemba and Rutowski [37]. We measured the eye surface area and the length of five rows of five facets each in four regions of the eye (i.e. ventral, dorsal, frontal and lateral regions). Digital pictures of the corneas were taken with a Canon EOS 5D Mark II camera mounted on a Nikon Eclipse 50i binocular microscope (Magnification: x4 for Fig 2b; Magnification: x20 for Fig 2c). For all image analyses, we used ImageJ software (v. 1.43u; [38]).

For the individuals of Group2, we measured thorax length (TL) along the dorsal anterior-posterior midline, thorax width (TW) at the widest part when viewed dorsally, and length of the leading edge of forewing (FW) using callipers. To do so, we immobilized the butterfly with open wings in the butterfly net, then blocked the left wing between the thumb and the annular and the right wing between the index and middle fingers inside the net, and finally took the butterfly out of the net and measured TL and TW. Next, the butterfly was released in the net, immobilized with closed wings, seized by the body between the thumb and the index finger and taken out of the net to measure FW. From these measures, we computed the volume of the thorax (TV), approximated as an ellipsoid volume with height equal to width: $TV=\frac{4}{3}\Pi \times {\left(\frac{TW}{2}\right)}^{2}\times \frac{TL}{2}$ and the wing loading (*WL* = *TV*/*FW*). Note that there are different ways to score the key components of the ratio that expresses wing loading, i.e. body mass and wing size, even within the Lepidoptera. Body mass has been expressed by, for example, body length (e.g. [39]), thorax length or width (e.g. [40]), thorax mass or total body mass (e.g. [15]) and wing size on the other hand by either forewing length (e.g. [39]), forewing area (e.g. [41]), total wing area of both fore and hindwings (e.g. [15]. Most of these traits are assumed to be correlated, but to the best of our knowledge, the impact of such differences for calculating wing loading has not been tested yet, but may hinder direct quantitative comparisons between studies.

### Interspecific comparison—Group1

First, we analysed the relationship between body size (forewing length) and the surface area of the eye using a two-way ANOVA that included the effects of species, sex and species by sex interaction (GLM procedure in SAS 9.3, www.sas.com). Second, we tested the allometric relationship between forewing length and eye area using Pearson correlation tests for each species and sex separately (CORR procedure in SAS). Finally, we compared the size of the facets using a mixed model implemented with a Gauss–Hermite quadrature estimation method (GLIMMIX procedure in SAS; [42,43]) with species, sex, eye region and their two- and three-way interactions as fixed effects. Individual was added as a random intercept in the model. There were several possible sources of error in the measurement (such as region assignment of facet diameter). Therefore, we removed from the analysis those measures of facet diameter (in an eye region for an individual) that deviated more than 1 SD from the mean, for that eye region and individual. This procedure left 3 to 5 replicate measures per eye region and individual.

### Interspecific comparison—Group2

Differences in thorax volume, wing length and wing loading were tested using two-way ANOVA models that included the effects of species, sex and species x sex interaction (GLM procedure in SAS).

### Intraspecific comparison: *B*. *eunomia* dispersers vs residents

The comparison between traits of residents and dispersers at the intraspecific level was limited to *B*. *eunomia*; indeed, no confirmed dispersers were observed for *B*. *aquilonaris* despite intensive MRR efforts (see below). First, we summarized the data collected on facet diameter in the four regions using a Principal Component Analysis (PRINCOMP procedure in SAS). The first composite axis (FACET; explaining 42% of the variance in the data) was positively correlated with the size of the facets in all eye regions. The second composite axis (FRONT; explaining 23% of the variance in the data) was positively correlated with the size of the facets in the frontal region only. Second, the differences between sexes for each measure (forewing length, eye area, FACET and FRONT) were removed by standardizing measures to get a mean of zero for each sex (STANDARD procedure in SAS). Finally, we used logistic regression models to test whether being a disperser or resident depended on these four variables (GENMOD procedure in SAS, with a logit link and a binomial distribution). We computed the 15 models formed by all the combinations of the four variables and we selected the model that predicted the status of dispersers with the lowest classification error rate, while using the lower number of variables (i.e. following the parsimony rule [44]). Instead of focusing on statistical significance for effect testing, we chose to focus on the ability of the models to correctly predict the status for observed dispersers. The rationale for using such an analysis is as follows. The status of dispersers was clear as we observed those individuals moving from one population to another, whereas the status of residents was more ambiguous, because it actually encompasses both real residents and potential, but unmotivated, dispersers that we observed for a long period in the same population. This implies that the difference in morphology between dispersers and residents will likely be less significant than in reality because variation in morphology in the resident group will overlap the variation in morphology in the disperser group. This is analogous to presence/absence data where presences are easily confirmed by the encounter of at least one individual, whereas absences can only be inferred with some level of probability (e.g. [45,46]).

## Results

### Specimen collection

We marked (and recaptured) 2262 (1735) individuals of *B*. *eunomia* and 3264 (827) of *B*. *aquilonaris*. For group 1, we collected 64 butterflies of the two species: 38 *B*. *eunomia* (20 females and 18 males) and 26 *B*. *aquilonaris* (10 females and 16 males). Among the *B*. *eunomia* individuals collected, we classified the dispersal status of 24 individuals: 11 were dispersers (5 females and 6 males dispersing 2037 m on average, range: 1172 m– 2708 m) and 13 were residents (8 females and 5 males staying in the habitat patch for 16 days on average, range: 10–19 days). The 14 other collected *B*. *eunomia* individuals were unclassified as they moved between very close patches belonging to the same genetic unit (unpublished data, average between patch distance: 444 m, range: 389–499 m), or they stayed (were recaptured) in the site of initial capture for too short of a period (4.7 days on average, range: 4–5 days) to reach sufficient confidence level they were residents. Although *B*. *aquilonaris* dispersers have been observed in previous years, none of the 3264 marked individuals were observed moving between populations most likely due to unsuitable weather conditions experienced during the flight season. Hence, no *B*. *aquilonaris* individuals could be classified as a disperser in this species this year.

For group 2, at the beginning of the flight period, we measured 482 live butterflies across all the populations, out of which 203 were *B*. *eunomia* (95 females and 108 males) and 279 were *B*. *aquilonaris* (139 females and 140 males).

### Interspecific comparison—Group1

**Fig 3:**
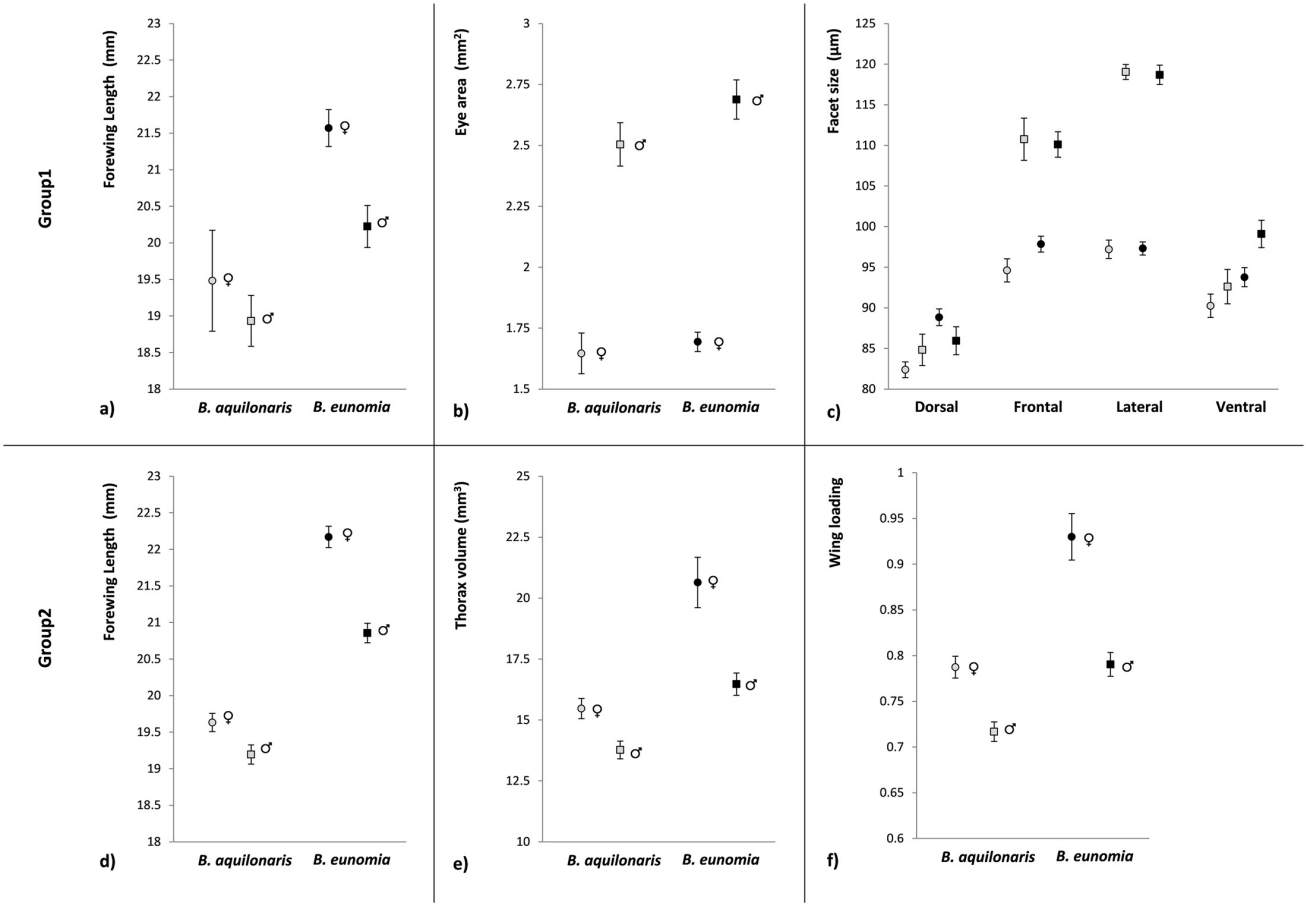
Differences in forewing length (a), eye area (b), and facet size by eye region (c) for Group 1 individuals and forewing length (d), thorax volume (e) and wing loading (f) for Group 2 individuals. Means and ± 95% confidence intervals are given for each species (grey symbols: *B*. *aquilonaris*; black symbols: *B*. *eunomia*), sexes (circles: females; squares: males) and eye region (dorsal, frontal, lateral and ventral) in (c). Raw data are available in S1 Table for Group 1 individuals and S2 Table for Group 2 individuals.

Overall, females had longer wings than males in both species and *B*. *eunomia* individuals were on average larger than *B*. *aquilonaris* in both sexes (Table 1a; Fig 3a). The sexual size dimorphism was smaller in *B*. *aquilonaris* compared to *B*. *eunomia*. Eye area was larger in males compared with females, with a nearly significant tendency to a larger sexual difference in *B*. *eunomia* (Table 1b; Fig 3b). Eye area was significantly positively correlated with forewing length in *B*. *aquilonaris* males only (*B*. *aquilonaris* females: r = 0.45, *P* = 0.23; *B*. *aquilonaris* males: r = 0.65, *P* = 0.004; *B*. *eunomia* females: r = -0.07, *P* = 0.77; *B*. *eunomia* males: r = 0.28, *P* = 0.25).

**Table 1:** Results of two-way ANOVAs (a-b, d-f) and mixed model (c) performed on morphological measurements.

**Effect**	**a) Forewing length *Group1***				**b) Eye area *Group1***				**c) Facet size *Group1***			
	**df num**	**df res**	***F***	***P***	**df num**	**df res**	***F***	***P***	**df num**	**df res**	***F***	***P***
Species	1	60	99.51	<.0001	1	60	10.16	0.0023	1	812	10.65	0.0011
Sex	1	60	31.27	<.0001	1	60	647.87	<.0001	1	812	168.15	<.0001
Species*Sex	1	60	5.52	0.0221	1	60	3.56	0.0641	1	812	1.17	0.2792
Region	/	/	/	/	/	/	/	/	3	812	375.65	<.0001
Species*Region	/	/	/	/	/	/	/	/	3	812	5.05	0.0018
Sex*Region	/	/	/	/	/	/	/	/	3	812	98.52	<.0001
Species*Sex*Region	/	/	/	/	/	/	/	/	3	812	1.92	0.1245
	**d) Forewing length *Group2***				**e) Thorax volume *Group2***				**f) Wing loading *Group2***			
	**df num**	**df res**	***F***	***P***	**df num**	**df res**	***F***	***P***	**df num**	**df res**	***F***	***P***
Species	1	478	901.77	<.0001	1	478	179.89	<.0001	1	478	64.06	<.0001
Sex	1	478	120.82	<.0001	1	478	87.56	<.0001	1	478	56.68	<.0001
Species*Sex	1	478	61.42	<.0001	1	478	26.68	<.0001	1	478	10.80	0.0011

Two-way ANOVAs including the effects of species, sex and species x sex interaction were used for forewing length in Group1 (a), eye area in Group1 (b), forewing length in Group2 (d), thorax volume in Group2 (e) and wing loading in Group2 (f). A mixed model including species, sex, eye region and their two- and three-way interactions as fixed effects was used for the of the facet size in Group1 (c). In this case, individual was added as a random intercept in the model.

Facets were larger i) over all eye regions and sexes in *B*. *eunomia* than in *B*. *aquilonaris*, ii) over all eye regions and species in males than in females, and iii) they decreased in size for all species and sexes from the lateral, frontal, ventral to the dorsal regions of the eye (Table 1c; Fig 3c). The sexual difference in facet size was significantly larger in *B*. *aquilonaris* compared with *B*. *eunomia*. In both species, facets were clearly smaller in the dorsal region and similar in the frontal and lateral regions. However, facets in the ventral region were more similar to those of the frontal and lateral regions in *B*. *eunomia* and to those of the dorsal region in *B*. *aquilonaris*. In males, facets clearly differed in size between regions as they gradually decreased from the lateral, frontal, ventral to dorsal regions of the eye. However in females, facets of the frontal and lateral regions were all of similar size. In summary: 1) male facets were of similar size in all regions while comparing species, except for the ventral region (smaller facets in *B*. *aquilonaris*) and decreased from lateral, frontal, ventral to dorsal regions of the eye; 2) female facets were larger in all regions for *B*. *eunomia* compared with *B*. *aquilonaris*, except in the lateral region; and 3) the increase in facet size in the frontal and lateral regions was more pronounced in males than in females of both species.

### Interspecific comparison—Group2

*B*. *eunomia* had longer wings and larger thoraxes than *B*. *aquilonaris* (Table 1d and 1e; Fig 3d and 3e). Females had longer wings and larger thoraxes than males for both species, but with a more pronounced sex difference in *B*. *eunomia* (Table 1d and 1e; Fig 3d and 3e). Wing loading was higher in *B*. *aquilonaris* than in *B*. *eunomia*, and in females than in males for both species, with a more pronounced sex difference in *B*. *aquilonaris* (Table 1f; Fig 3f).

### Intraspecific comparison: *B*. *eunomia* dispersers vs. residents

**Fig 4:**
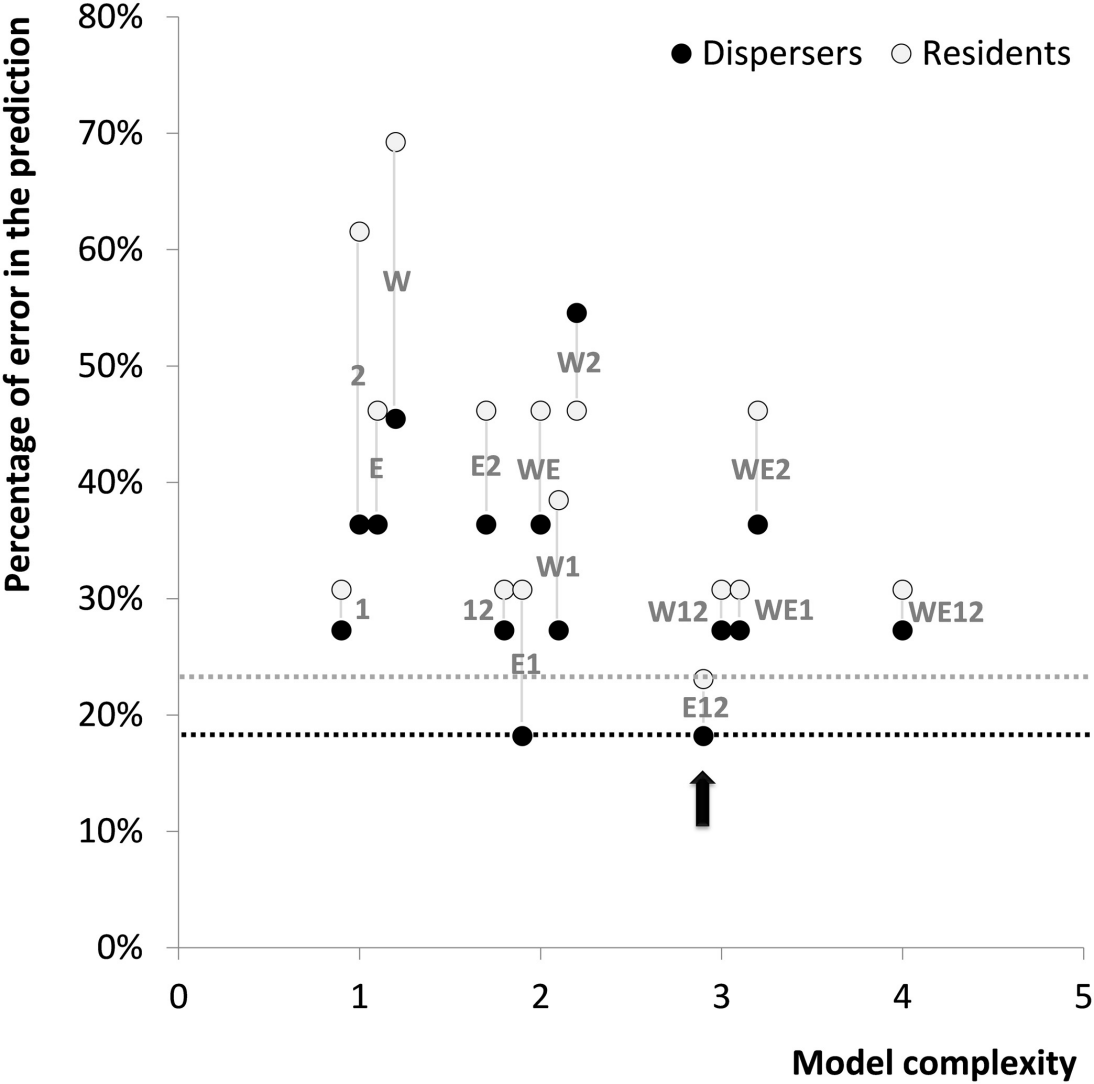
Model performance using morphological variables for predicting the individual dispersal status according to model complexity. Each dot represents the result of one of the 14 tested models (the null model was not included). The individual dispersal status corresponded to disperser *vs*. resident (Disperser: black circle *vs*. resident: grey circles). Model complexity is expressed as the number of explanatory variables used (Forewing length: W, Eye area: E, FACET: 1 and FRONT: 2). Black dotted line: smallest error rate for the prediction of dispersers. Grey dotted line: smallest error rate for the prediction of residents. Black arrow: the model selected as best because it combined lower prediction error for both dispersers and residents.

**Fig 5:**
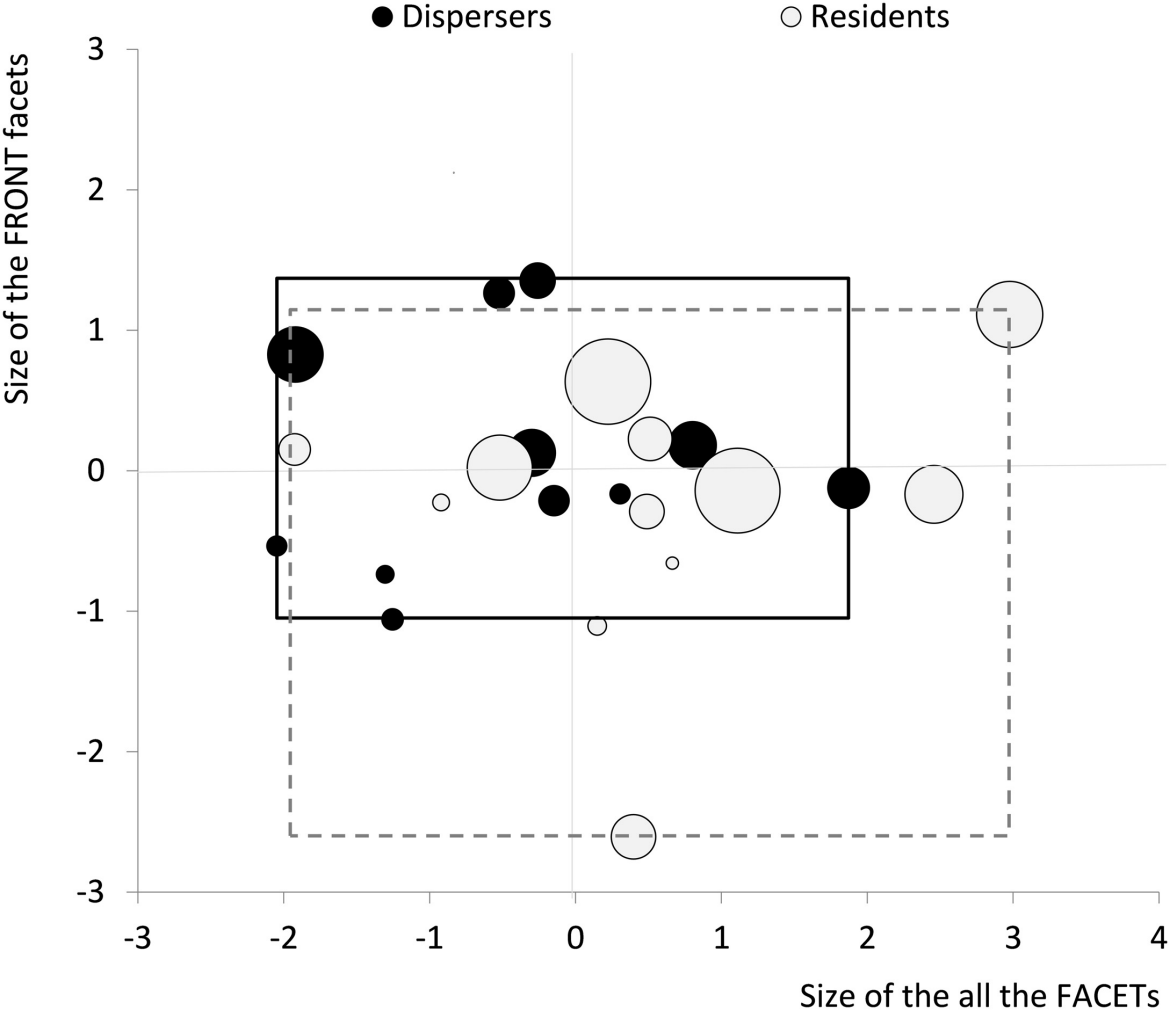
Variation in eye morphology in relation to the dispersal status of *B*. *eunomia* individuals. Dispersers: black circles within the black rectangle *vs*. residents: grey circles within the dotted grey rectangle. Eye area is translated in the circle size. Dispersers tended to have smaller eyes than residents with smaller facets (FACET: X-axis, corresponding to an increasing size of all facets, as summarised by the first axis of the Principal Component Analysis), except in the frontal region where facets were actually larger (FRONT: Y-axis, corresponding to an increasing size of the facets in the frontal region of the eyes, as summarised by the second axis of the Principal Component Analysis).

The best predictor of dispersal status (disperser vs. resident) for *B*. *eunomia* individuals was obtained by a logistic regression model including three out of the four morphological variables (eye area, FACET and FRONT; Fig 4). Comparing models of increasing complexity, we could rank the relative predictive power of these three variables as FACET > eye area > FRONT. Accordingly, dispersers tended to have smaller eyes than residents (around 2% smaller, Eye area model estimate = -0.28; Fig 5) with smaller facets (around 1.5% smaller, FACET model estimate = -0.46; Fig 5), except in the frontal region where facets were actually larger (around 30% larger, FRONT model estimate = 0.21; Fig 5).

## Discussion

### Sexual dimorphism in flight and compound eye morphology

In butterflies, adult males and females perform a number of similar activities (e.g. nectar feeding), but there are clearly sex-specific behaviours (e.g. mate locating for males vs. searching for oviposition sites for females). These behavioural differences are expected to produce sexual differences in the demands on both visual and flight systems.

In both species, females had larger wings and thoraxes than males confirming our earlier analyses for these species [32], as well as for nymphalid species in general [47]. This suggests an increased allocation to the flight apparatus (i.e. muscles in the thorax and larger wing area) in females presumably to maintain flight performance with heavy egg loads. Wing loading can influence both the flight velocity and acceleration [15] (but see [48]). As in flies [49] and other butterfly species [50], males had larger compound eyes than females in both our study species. In butterflies, males may have eyes that are up to 30% larger than those of females [28].

In both sexes, the facets in the lateral area of the eyes were largest, and in males of both species, the frontal area of the eyes also had relatively large facets. It has been suggested that a frontal acute zone in males is adaptive in the context of recognizing and tracking flying conspecific females (e.g. as in the butterfly *Colias eurytheme;* [26], and in flies; [49]). In females, the search for appropriate egg laying sites is mainly driven by chemical recognition, but visual signals may play a role as well (e.g. [51,52]). The frontal and ventrofrontal parts of the eyes are expected to be adapted for detection and recognition of host plants [53]. Accordingly, in females of *B*. *eunomia* and *B*. *aquilonaris*, facets in the frontal and ventral areas were relatively large compared with the dorsal area.

### Intraspecific variation: dispersers have smaller eyes and larger frontal ommatidia than residents

Our results with butterfly eyes not only support the expectations from sexual differences in the visual tasks described above, but also suggest key role of vision in navigating in the environment. Therefore, the frontal and ventral areas of the eyes are also expected to be acute zones. Indeed, according to Land and Eckert [49], “the anterior direction represents the animal’s immediate future […]. If there are obstacles ahead, then they can be detected at greater distances if the acuity is high in this region”. A greater acuity and/or sensitivity in the frontal region should help getting information about important features and objects in the environment [54].

In *B*. *eunomia*, dispersers had smaller eyes, with smaller facets in general, but larger facets in the frontal region than residents. Hence, individuals that dispersed successfully have a potentially increased visual acuity in the frontal region. This could improve their ability to direct their flight to target habitats or specific vegetation features at a distance or to landmarks such as rivers that would facilitate movement to the next habitat patch. Also, it could indicate a trade-off between “brain-power” allocated to navigation vs. other cognitive processes, or an energetic trade-off, as information processing is metabolically costly. Perceptual range (i.e. the distance at which individuals perceive their habitat) was estimated at < 30 m in this species [55]; such an estimation was made possible because the habitat is structured as discrete spatial entities with sharp boundaries. Although we detected differences in the visual system, we did not detect any difference in wing size between dispersers and residents suggesting that dispersers and residents may have similar motion capacities in this study landscape, but different dispersal-related navigation capacities.

Some potentially important factors were not controlled or measured in our study. First, the motivation to disperse was not considered. Hence, individuals classified as residents may have been unmotivated, potential dispersers who simply had not been subject to the right stimuli and / or conditions to trigger dispersal. Second, we were obviously unable to detect, hence collect, marked individuals who may have emigrated but did not succeed in reaching another habitat patch. These factors may have lowered our ability to detect strong morphological differences between dispersers and residents. Further experiments, under controlled semi-natural conditions (e.g. [56]) are now required to further explore the observed relationships. An interesting alternative or complementary approach consists of comparing flight and eye morphology in a larger sample of butterflies from different landscapes with contrasted levels of habitat fragmentation.

### Interspecific variation: influence of habitat distribution

Species-specific patterns of habitat distribution and associated selection on dispersal may have shaped the evolution of interspecific differences in flight and eye morphology in butterflies. As mentioned previously, habitat patches of *B*. *eunomia* are relatively continuous in our study system while bogs inhabited by *B*. *aquilonaris* have been fragmented in Belgium for decades. Consequently, dispersal distances are on average greater in *B*. *aquilonaris* than in *B*. *eunomia*.

Despite *B*. *eunomia* individuals having larger wings and bigger thoraxes, *B*. *aquilonaris* individuals had higher wing loading. Therefore, our results support the evolutionary response towards long-distance dispersal flights under fragmented habitat distribution. Higher wing loading is expected to confer better flight performance in terms of flight speed [15]. However, as already indicated in the introduction, the expected relationship between wing loading and dispersal is not clear and will depend on those components of flight performance (e.g. speed vs. endurance) that are most important to successful between-habitat patch movements. Having a lower wing loading may mean lower energetic cost for flight for dispersers, but whether this tactic is evolutionary stable may depend on overall energy budgets, including trade-offs with other life history traits that affect fitness (e.g. fecundity). Insects’ flight performance will also be a function of wing kinematics (i.e. wing stroke amplitude, stroke frequency and the force coefficient of their wings) which is not reflected by simple wing loading measures [57]). Further study will be needed to sort out these complex connections between dispersal, body structure, and fitness.

Despite having smaller body and wing sizes, *B*. *aquilonaris*, which has previously been found to be more dispersive than *B*. *eunomia*, has larger eyes, and larger facets at least in the frontal and lateral eye regions than *B*. *eunomia*. Eye formation and neural processing of information gathered by the eyes are metabolically costly [58–61], which means *B*. *aquilonaris* makes a larger investment in their visual system. This higher relative investment by *B*. *aquilonaris* compared with *B*. *eunomia* may have evolved specifically to facilitate the detection of the fragmented habitat patches from a distance. In line with this finding, note that in *B*. *aquilonaris* the perceptual range is > 60 m, which is more than twice the perceptual range of *B*. *eunomia* (unpublished results). However, the distance at which such habitat patches can be detected will depend on the sensory ‘signatures’ of the habitat target (i.e. the variety of cues used which might include olfactory as well as visual cues; [12]) and on the sensitivities of the relevant sensory systems. Empirical studies of the precise nature and relative importance of these cues will be needed to better understand the evolved relationships between dispersal tactics and sensory systems.

## Conclusions

Dispersal ecology and evolution are areas of recent active study, but there are still significant gaps in our mechanistic understanding. For many study species we lack detailed information on the role of behavioural, morphological and physiological traits for the dispersal process. Such information is difficult to extract from data based on multi-site Mark-Release-Recapture protocols or analyses of gene flow. Tracking individuals and recording their responses to biotic and abiotic variables is the best, though labour-intensive, approach to get insights into the behavioural processes at work during dispersal. Although morphology has attracted attention, the exploration of the sensory and neurobiological mechanisms is also crucial to fully comprehend the dispersal process, but far less investigated. An instructive exception is the work on insect migration (e.g. migratory Monarch butterflies; [62]). To go beyond the short cut of correlative analysis between morphology and behaviour, we need an analytical approach of the process linking compound eye properties and specialisations to vision and navigation such as, for example, in flies [63–65]. Therefore, our novel study suggests interesting paths for future research in the sensory ecology of dispersal [66].

## Supporting Information

S1 Table Individual measures of forewing length (mm), eye area (mm^2^), and facet size (μm) by eye region (dorsal, frontal, lateral and ventral) for Group 1 individuals. Species: *Boloria eunomia* and *B*. *aquilonaris*. ID: individual identifier. Group: disperser, resident or unclassified (See methods for a description). Sex: F = female, M = male.
(XLSX)

S2 Table Individual measures of forewing length (mm), thorax length (mm), and thorax width (mm) for Group 2 individuals. Species: *Boloria eunomia* and *B*. *aquilonaris*. ID: individual identifier. Population: population of origin. Sex: F = female, M = male.
(XLSX)

## References

[1] Ronce O. A does it feel to be like a rolling stone? Ten questions about dispersal evolution. Annu Rev Ecol Evol Syst. 2007;38: 231–253.

[2] Baguette M, Blanchet S, Legrand D, Stevens VM, Turlure C. Individual dispersal, landscape connectivity and ecological networks. Biol Rev. 2013;88: 310–326. 10.1111/brv.12000 23176626

[3] Blanchet S, Rey O, Etienne R, Lek S, Loot G. Species-specific responses to landscape fragmentation: implications for management strategies. Evol Appl. 2010;3: 291–304. 10.1111/j.1752-4571.2009.00110.x 25567925 PMC3352461

[4] Hanski I. Landscape fragmentation, biodiversity loss and the societal response. EMBO reports. 2005;6: 388–392. 15864284 10.1038/sj.embor.7400398 PMC1299308

[5] Bohonak AJ. Dispersal, gene flow, and population structure. The Quarterly Review of Biology. 1999;74: 21–45. 10081813 10.1086/392950

[6] Levin SA, Cohen D, Hastings A. Dispersal strategies in patchy environments. Theoretical Population Biology. 1984;26: 165–191.

[7] Nathan R, Getz WM, Revilla E, Holyoak M, Kadmon R, Saltz D, et al A movement ecology paradigm for unifying organismal movement research. Proc Nat Ac Sciences. 2008;105: 19052–19059. 10.1073/pnas.0800375105 PMC2614714 19060196

[8] Baguette M, Stevens V, Clobert J. The pros and cons of applying the movement ecology paradigm for studying animal dispersal. Movement Ecology. 2014;2: 13.

[9] Bowler DE, Benton TG. Causes and consequences of animal dispersal strategies: relating individual behaviour to spatial dynamics. Biol Rev. 2005;80: 205–225. 15921049 10.1017/s1464793104006645

[10] Matthysen E. Density-dependent dispersal in birds and mammals. Ecography. 2005;28: 403–416.

[11] Holyoak M, Casagrandi R, Nathan R, Revilla E, Spiegel O. Trends and missing parts in the study of movement ecology. Proc Nat Ac Sciences. 2008;105: 19060–19065. 10.1073/pnas.0800483105 PMC2614715 19060194

[12] Kinoshita M, Shimohigasshi M, Tominaga Y, Arikawa K, Homberg U. Topographically distinct visual and olfactory inputs to the mushroom body in the Swallowtail butterfly, *Papilio xuthus*. Journal of Comparative Neurology. 2015;523: 162–182. 10.1002/cne.23674 25209173

[13] Dingle H. Migration The biology of life on the move. New York: Oxford University Press; 1996.

[14] Stevens VM, Trochet A, Van Dyck H, Clobert J, Baguette M. How is dispersal integrated in life histories: a quantitative analysis using butterflies. Ecol Lett. 2012;15: 74–86. 10.1111/j.1461-0248.2011.01709.x 22070676

[15] Betts CR, Wootton RJ. Wing shape and flight behaviour in butterflies (Lepidoptera Papilionidae and Hesperioidiae): a preliminary analysis. J Exp Biol. 1988;138: 271–288.

[16] Wickman PO. Sexual selection and butterfly design—a comparative study. Evolution. 1992;46: 1525–1536. 28568994 10.1111/j.1558-5646.1992.tb01142.x

[17] Weber JM. The physiology of long-distance migration: extending the limits of endurance metabolism. J Exp Biol. 2009;212: 593–597. 10.1242/jeb.015024 19218508

[18] Merlin C, Gegear RJ, Reppert SM. Antennal circadian clocks coordinate sun compass orientation in migratory Monarch butterflies. Science. 2009;325: 1700–1704. 10.1126/science.1176221 19779201 PMC2754321

[19] Harrison RG. Dispersal Polymorphisms in Insects. Annu Rev Ecol Syst. 1980;11: 95–118.

[20] Meylan S, de Fraipont M, Aragon P, Vercken E, Clobert J. Are dispersal-dependent behavioral traits produced by phenotypic plasticity? J Exp Zool. 2009;311A: 377–388. 10.1002/jez.533 19350544

[21] Parmesan C. Ecological and evolutionary responses to recent climate change. Annu Rev Ecol Evol Syst. 2006;37: 637–669.

[22] Altizer S, Davis AK. Populations of Monarch butterflies with different migratory behaviors show divergence in wing morphology. Evolution. 2010;64: 1018–1028. 10.1111/j.1558-5646.2010.00946.x 20067519

[23] Berwaerts K, Aerts P, Van Dyck H. On the sex-specific mechanisms of butterfly flight: flight performance relative to flight morphology, wing kinematics, and sex in *Pararge aegeria*. Biol J Linn Soc. 2006;89: 675–687.

[24] Hill JK, Thomas CD, Blakeley DS. Evolution of flight morphology in a butterfly that has recently expanded its geographic range. Oecologia. 1999;121: 165–170. 28308556 10.1007/s004420050918

[25] Niitepõld K, Smith AD, Osborne JL, Reynolds DR, Carreck NL, Martin AP, et al Flight metabolic rate and Pgi genotype influence butterfly dispersal rate in the field. Ecology. 2009;90: 2223–2232. 19739384 10.1890/08-1498.1

[26] Merry JW, Morehouse NI, Yturralde K, Rutowski RL. The eyes of a patrolling butterfly: Visual field and eye structure in the Orange Sulphur, *Colias eurytheme* (Lepidoptera, Pieridae). J Insect Physiol. 2006;52: 240–248. 16360167 10.1016/j.jinsphys.2005.11.002

[27] Rutowski R, Warrant EJ. Visual field structure in the Empress Leilia, *Asterocampa leilia* (Lepidoptera, Nymphalidae): dimensions and regional variation in acuity. J Comp Physiol A. 2002;188: 1–12. 10.1007/s00359-001-0273-7 11935226

[28] Rutowski RL. Variation of eye size in butterflies: inter- and intraspecific patterns. J Zool. 2000;252: 187–195.

[29] Turlure C, Baguette M, Stevens VM, Maes D. Species- and sex-specific adjustments of movement behavior to landscape heterogeneity in butterflies. Behav Ecol. 2011;22: 967–975.

[30] Nève G, Barascud B, Hughes R, Aubert J, Descimon H, Lebrun P, et al Dispersal, colonization power and metapopulation structure in the vulnerable butterfly *Proclossiana eunomia* (Lepidoptera: Nymphalidae). J Appl Ecol. 1996;33: 14–22.

[31] Baguette M. Long distance dispersal and landscape occupancy in a metapopulation of the cranberry fritillary butterfly. Ecography. 2003;26: 153–160.

[32] Turlure C, Schtickzelle N, Baguette M. Resource grain scales mobility and adult morphology in butterflies. Land Ecol. 2010;25: 95–108.

[33] Van Dyck H. Dispersal under global change—The case of the Processionary moth and other insects In: Baguette M, Benton TG, Bullock JM, editors. Dispersal Ecology and Evolution. Oxford: Oxford University Press; 2012 pp. 357–365.

[34] Almbro M, Kullberg C. Weight loading and reproductive status affect the flight performance of *Pieris napi* butterflies. J Insect Behav. 2012;25: 441–452.

[35] Duthie AB, Abbott KC, Nason JD. Trade-offs and coexistence in fluctuating environments: evidence for a key dispersal-fecundity trade-off in five nonpollinating fig wasps. Am Nat. 2015;186: 151–158. 10.1086/681621 26098346

[36] Turlure C, Choutt J, Van Dyck H, Baguette M, Schtickzelle N (2010) Functional habitat area as a reliable proxy for population size: case study using two butterfly species of conservation concern. J Insect Conserv.

[37] Ziemba K, Rutowski RL. Sexual dimorphism in eye morphology in a butterfly (*Asterocampa leilia*; Lepidoptera, Nymphalidae). Psyche. 2000;103: 25–36.

[38] Schneider CA, Rasband WS, Eliceiri KW. NIH Image to ImageJ: 25 years of image analysis. Nature Methods. 2012;9: 671–675. 22930834 10.1038/nmeth.2089 PMC5554542

[39] Dantanarayana W. Environmentally cued size variation in the light-brown apple moth,*Epiphyas postvittana* (Walk.) (Tortricidae), and its adaptive value in dispersal. Oecologia. 1976;26: 121–132. 28309256 10.1007/BF00582890

[40] Barker JSF, Krebs RA. Genetic variation and plasticity of thorax length and wing length in *Drosophila aldrichi* and *D*. *buzzatii*. Journal of Evolutionary Biology. 1995;8: 689–709.

[41] Berwaerts K, Van Dyck H, Aerts P. Does flight morphology relate to flight performance? An experimental test with the butterfly *Pararge aegeria*. Funct Ecol. 2002;16: 484–491.

[42] Bolker BM, Brooks ME, Clark CJ, Geange SW, Poulsen JR, Stevens HH, et al Generalized linear mixed models: a practical guide for ecology and evolution. Trends Ecol Evol. 2009;24: 127–135. 10.1016/j.tree.2008.10.008 19185386

[43] Zuur AF, Leno EN, Walker N. Mixed effects models and extensions in ecology with R. New York, USA: Springer; 2009.

[44] Burnham KP, Anderson DR. Model selection and multimodel inference: a practical information-theoretic approach. New York: Springer-Verlag; 2002.

[45] Ry M. Inferring the absence of a species: A case study of snakes. J Wildlife Manage. 2002;66: 330–338.

[46] Gu W, Swihart RK. Absent or undetected? Effects of non-detection of species occurrence on wildlife-habitat models. Biol Conserv. 2004;116: 195–203.

[47] Shreeve TG, Konvicka M, Van Dyck H. The functional significance of butterfly wing morphology variation In: Settele J, Shreeve TG, Konvicka M, Van Dyck H, editors. Ecology of Butterflies in Europe. Cambridge: Cambridge University Press; 2009 pp. 171–188.

[48] Srygley RB, Kingsolver J. Effects of weight loading on flight performance and survival of palatable Neotropical *Anartia fatima* butterflies. Biol J Linn Soc. 2000;70: 707–725.

[49] Land MF, Eckert H. Maps of the acute zones of fly eyes. J Comp Physiol. 1985;156: 525–538.

[50] Yagi N, Koyama N. The compound eye of lepidoptera: approach from organic evolution. Tokyo: Shinkyo Press; 1963.

[51] Garcia-Barros E, Fartmann T. Butterfly oviposition: sites, behaviour and modes In: Settele J, Shreeve TG, Konvicka M, Van Dyck H, editors. Ecology of butterflies in Europe. Cambridge: Cambridge University Press; 2009 pp. 29–42.

[52] Prokopy RJ, Owens ED. Visual detection of plants by herbivorous insects. Annu Rev Entomol. 1983;28: 337–364.

[53] Rutowski RL. Visual ecology of adult butterflies In: Boggs CL, Watt WB, Ehrlich PR, editors. Ecology and evolution taking flight: Butterflies as model study systems. Chicago: University of Chicago Press; 2003 pp. 9–25.

[54] Land MF, Nilsson DE. General-purpose and special-purpose visual systems In: Warrant EJ, Nilsson DE, editors. Invertebrate vision. Cambridge: Cambridge University Press; 2006 pp. 167–210.

[55] Schtickzelle N, Joiris A, Van Dyck H, Baguette M. Quantitative analysis of changes in movement behaviour within and outside habitat in a specialist butterfly. BMC Evol Biol. 2007;7: 4 17241457 10.1186/1471-2148-7-4 PMC1784076

[56] Ducatez S, Legrand D, Chaput-Bardy A, Stevens VM, Freville H, Baguette M. Inter-individual variation in movement: is there a mobility syndrome in the large white butterfly Pieris brassicae? Ecol Entomol. 2012;37: 377–385.

[57] Lehmann FO, Dickinson MH. The control of wing kinematics and flight forces in fruit flies (Drosophila spp.). J Exp Biol. 1998;201: 385–401. 9427672 10.1242/jeb.201.3.385

[58] Horridge A. Visual processing of pattern In: Warrant EJ, Nilsson DE, editors. Invertebrate vision. Cambridge: Cambridge University Press; 2006 pp. 494–523.

[59] Laughlin SB, de Ruyter van Steveninck R, Anderson JC. The metabolic cost of neural information. Nat Neurosci. 1998;1: 36–41. 10195106 10.1038/236

[60] Niven JE, Anderson JC, Laughlin SB. Fly photoreceptors demonstrate energy-information trade-offs in neural coding. 2007;5: 828–840. 10.1371/journal.pbio.0050116 PMC1828148 17373859

[61] Niven JE, Laughlin SB. Energy limitation as a selective pressure on the evolution of sensory systems. 2008;211: 1792–1804. 10.1242/jeb.017574 18490395

[62] Merlin C, Heinze S, Reppert SM. Unraveling navigational strategies in migratory insects. Current Opinion in Neurobiology. 2012;22: 353–361. 10.1016/j.conb.2011.11.009 22154565 PMC3306460

[63] Burton BG, Tatler BW, Laughlin SB. Variations in photoreceptor response dynamics across the fly retina. Journal of Neurophysiology. 2001;86: 950–960. 11495963 10.1152/jn.2001.86.2.950

[64] Hornstein EP, Carroll DC, Anderson JC, Laughlin SB. Sexual dimorphism matches photoreceptor performance to behavioural requirements. Proceedings of the Royal Society of London B: Biological Sciences. 2000;267: 2111–2117. 10.1098/rspb.2000.1257 PMC1690788 11416917

[65] Nordström K, Barnett PD, Moyer de Miguel IM, Brinkworth RSA, O'Carroll DC. Sexual dimorphism in the hoverfly motion vision pathway. Current Biology. 2008;18: 661–667. 10.1016/j.cub.2008.03.061 18450449

[66] Greggor AL, Clayton NS, Phalan B, Thornton A. Comparative cognition for conservationists. Trends Ecol Evol. 2014;29: 489–495. 10.1016/j.tree.2014.06.004 25043737 PMC4153814

